# Factors Affecting Pain in Hemodialysis and Non-pharmacological Management

**DOI:** 10.7759/cureus.35448

**Published:** 2023-02-25

**Authors:** Georgia Gerogianni

**Affiliations:** 1 Department of Nursing, University of West Attica, Athens, GRC

**Keywords:** causes, management, non-pharmacological treatments, hemodialysis, pain

## Abstract

Pain is a frequent problem among hemodialysis patients, mostly caused by painful procedures, acute complications of hemodialysis, and painful syndromes, such as musculoskeletal and neuropathic syndromes. Pain can frequently lead to sleep disturbances, reduced adherence to hemodialysis treatment, frequent hospital admissions, decreased quality of life, and high mortality rates. Non-pharmacological management of pain in the hemodialysis population includes aerobic and resistance exercises, music therapy, and cognitive behavioral therapy. The present review focuses on the factors affecting pain in hemodialysis and its non-pharmacological management, offering important information to renal professionals.

## Introduction and background

Pain is a common problem among patients on hemodialysis, with incidence ranging from 8 to 82% [1]. It is mostly caused by painful procedures accompanying hemodialysis (i.e., venipuncture), surgery for the creation of vascular access [2,3], acute complications during hemodialysis regimen (i.e., muscle cramps, headaches) [2], painful syndromes, such as ischemic limbs, and musculoskeletal and neuropathic syndromes [1,4].

Muscle cramps are mostly caused by hypotension due to excessive fluid removal during hemodialysis and electrolyte disturbances [5]. Similarly, the use of central catheters during hemodialysis can cause painful neuropathies due to ischemia [6]. Additionally, air embolism is a serious complication during hemodialysis which can cause severe pain around the chest and lead to shortness of breath [7].

In a study with 300 patients on hemodialysis, it was found that pain had a significant association with patients’ comorbidities and increased body mass index. It can be assumed that comorbidities usually increase pain, while obesity possibly deteriorates chronic pain by reducing the pain threshold and increasing its sensitivity [8]. Similarly, in a study among 328 hemodialysis patients, pain had a significant association with the female gender, possibly because women tend to express their complaints more frequently than males and are more sensitive to pain due to mechanisms of peripheral and central perception systems [9].

Pain can negatively affect the mental health of dialysis patients [3] and can increase the levels of anxiety and depression [2,10]. It has been found that patients with chronic pain have more depressive symptoms than those without pain [11]. Additionally, inadequate pain management can cause sleep disturbances and reduced adherence to hemodialysis treatment [8,4]. Chronic pain is associated with frequent hospital admissions, decreased quality of life, and high mortality rates [1,12]. It has been found that a significant proportion of dialysis people have increased pain, both during dialysis and non-dialysis days [13].

## Review

Musculoskeletal pain in hemodialysis

Renal bone disease is a frequent problem in hemodialysis which is usually caused by disorders in calcium-phosphate metabolism, lack of vitamin D, and uremic toxins [14]. Hsu et al. [15] in their study of 456 patients with chronic kidney disease found that chronic musculoskeletal pain had a significant association with hyperuricemia and a high calcium-phosphate product.

Additionally, comorbid diseases such as ischemic peripheral artery disease, diabetic neuropathy, and osteopenia/osteoporosis frequently lead to different types of musculoskeletal pain [4]. In a study with 49 patients on hemodialysis, the most frequent rheumatic and musculoskeletal diseases were fibromyalgia syndrome (51%), myalgia (37%), arthralgia (37%), flexor tenosynovitis (29%), and cramps (29%) [16].

Additionally, long-term dialysis is related to amyloidosis because amyloid deposition occurs in musculoskeletal structures which clinically manifests as destructive arthropathy, erosive spondyloarthropathy, and bone cysts [16]. In a similar study involving 144 hemodialysis patients, it was found that 60.4% of the participants had musculoskeletal pain. The most frequent symptoms were joint pain (arthralgia) (25.3%), osteoarthritis (17.2%), carpal tunnel syndrome (14.9%), and osteoporosis (13.8%) [17]. A triad of shoulder periarthritis, carpal tunnel syndrome, and flexor tenosynovitis of hands has been identified in patients on long-term hemodialysis and peritoneal dialysis due to β2-microglobulin amyloid deposition [17].

Moreover, Molsted and Eidemak [18] found in their study of 539 patients with chronic kidney disease that two-thirds of the participants had musculoskeletal pain, which was related to low quality of life and low physical well-being. In a similar study, it was reported that pain in hemodialysis patients had a negative effect on their general activity, mobility, and mood because pain in the upper limb significantly affected patients’ capability to walk [4]. Similarly, in a study with 100 hemodialysis patients, 20.4% of the participants reported that pain had a negative effect on their usual work [19].

Restless leg syndrome

Restless leg syndrome is a disturbance of the central nervous system, wherein patients have the necessity to move their legs or other parts of their body while sleeping. A common symptom of restless leg syndrome is pain in the calves and legs, especially when the legs are inactive [20]. The main causes of restless leg syndrome are uremic toxins in the blood, lack of iron, and diabetic neuropathy [1]. In uremic restless leg syndrome, symptoms of muscle atrophy have been identified [21]. Dialysis patients with restless leg syndrome usually have high levels of fatigue [22] and sleep disorders [12].

Pain during fistula cannulation

About 20% of patients on hemodialysis have serious pain during cannulation, despite the use of analgesics topically [23]. About 48% of dialysis people are afraid of the pain associated with the insertion of needles into the fistula. The pain experienced by these individuals is mostly related to fistula puncture and can cause depressive symptoms [24].

Additionally, in patients with cardiovascular diseases, cardiac complications are significantly increased during and after the surgery for the creation of vascular access due to anxiety because anxiety raises heart and respiratory rates, cardiac output, and oxygen requirements [25]. In their study, Ibrahim et al. [7] reported that 32.5% of hemodialysis patients had moderate pain during fistula cannulation, while 56.4% of the respondents had varying levels of anxiety during fistula puncture. Moreover, these individuals usually have pain in the access arm between dialysis regimens [26]. Pain is one of the main causes of patients’ withdrawal from hemodialysis because they need about 150 hemodialysis regimens per year [23].

Non-pharmacological management of pain in hemodialysis

Effective management of pain in renal patients includes management of pain related to fistula puncture, aerobic and resistance exercises, music therapy, and cognitive behavioral therapy. Additionally, good sleeping conditions, reduction of the use of alcohol and caffeine, and kidney transplantation can lead to a reduction of symptoms of restless leg syndrome [21]. However, complex pain syndromes need analgesic drugs, including opioids and non-opioids, according to the needs of each patient [4].

Aerobic Exercises

Aerobic exercises improve the quality of life and reduce mortality rates in dialysis patients [21]. Regular exercise can reduce fatigue and musculoskeletal pain while strengthening muscles and improving physical and mental well-being [27]. In a study of 43 patients with chronic kidney disease who followed aerobic and resistance exercises on non-dialysis days, it was found that they had a significant improvement in quality of life parameters, such as physical function, body pain, general health, and vitality [28].

It has been found that aerobic exercises reduce different inflammatory cytokines and improve oxidative stress in patients with chronic kidney disease [29]. In a similar study among 90 patients on hemodialysis, it was found that stretching exercises had a positive effect on the reduction of restless leg syndrome [30]. Similarly, in a study of 50 elderly hemodialysis patients, there was a significant reduction in muscle cramps in participants who followed stretching exercises during hemodialysis [31].

Music Therapy

It has been found that music therapy can significantly decrease pain and anxiety during dialysis therapy [32] and is efficient for people with painful muscle cramps and musculoskeletal pain [24]. Additionally, Inayama et al. [23] in their study with 121 hemodialysis patients found that listening to music significantly reduced pain levels during cannulation. In a similar study, with 114 hemodialysis patients, it was found that self-selected soothing music relieved patients from pain caused by fistula puncture [24].

Similarly, in a study with 65 dialysis patients, it was shown that participants who were listening to music during vascular access surgery had significantly lower perceived levels of pain than the control group [33]. In a similar study with 55 patients who underwent fistula surgery, patients who listened to music during the fistula surgery had significantly lower anxiety and better heart rate, respiratory rate, and oxygen saturation than the control group [25]. During arteriovenous fistula surgery, music therapy distracts patients from stressful sounds, such as conversations in the operating room and orders of surgeons, thus improving the perceived pain and patients’ anxiety [25].

When people listen to their desired music, it helps them reduce the feeling of pain possibly because music affects the autonomic nervous system and leads to emotional and physiological improvement [33]. Music therapy can lead to hemodynamic changes, such as reduced heart rate and blood pressure [24], while it increases positive emotions and reduces negative emotions [34] Additionally, it acts as an analgetic because it releases dopamine and endorphins, which, in turn, balances the autonomic nervous system [34].

Thus, music therapy is a safe and cheap method, with no side effects of pharmacological therapy, and can be easily applied to reduce pain and anxiety during fistula punctures [35].

Cognitive Behavioral Therapy

Pain is not only a sensory but also an emotional experience [36]. Cognitive behavior therapy for chronic pain helps people increase their capability to control pain and acquire knowledge about coping skills that can decrease distress derived from pain. It also includes education about pain for people to realize that pain can be affected by behaviors, thoughts, and feelings. Additionally, it includes education in reframing unhelpful opinions about pain and relaxation methods to reduce stress. Finally, it includes the use of learned techniques in daily painful conditions [32]. Chen et al. [37], in a study with 72 dialysis patients with sleep disorders, found that those who followed cognitive behavioral therapy had an improvement in their sleep disorders and a decrease in inflammation and oxidative stress.

## Conclusions

Pain is a frequent problem among dialysis patients. Pain can frequently lead to sleep disturbances and reduced adherence to hemodialysis treatment, frequent hospital admissions, decreased quality of life, and high mortality rates. Non-pharmacological management of pain in the renal population includes management of pain related to fistula cannulation, aerobic and resistance exercises, music therapy, and cognitive behavioral therapy. However, complex pain syndromes need analgesic drugs tailored to the needs of each patient.
